# De Novo Hypercalcaemia in a Patient With Chronic Hypoparathyroidism

**DOI:** 10.7759/cureus.74923

**Published:** 2024-12-01

**Authors:** Babasola Sola-Oladokun, Muhammad Usman, Sean Manning

**Affiliations:** 1 Endocrinology, Mallow General Hospital/University College Cork, Cork, IRL; 2 Medicine, Mallow General Hospital/University College Cork, Cork, IRL

**Keywords:** 25 (oh) vitamin d, calcium metabolism, calcium therapy, pulmonary sarcoidosis, vitamin d toxicity

## Abstract

Calcium Homeostasis in the human body is regulated by hormones, including parathyroid hormone and vitamin D3. Dysfunction in the form of hypoparathyroidism causes hypocalcaemia. In patients treated for primary hypoparathyroidism with activated vitamin D replacement, iatrogenic hypercalcaemia can occur. This must be investigated to exclude other aetiologies, such as malignancy and granulomatous disease.

In this case report, we describe a 73-year-old man with a distant history of chronic hypoparathyroidism treated with vitamin D3 who presented with lethargy, confusion, polyuria, and polydipsia. On admission, he was found to be hypercalcaemic at 3.22 mmol/L. He presented on two previous occasions with symptomatic hypocalcaemia that resulted in a reduction and subsequent cessation of his vitamin D supplementation. This hypercalcaemia persisted, prompting investigations of non-iatrogenic causes. Computer tomography (CT) and positron emission tomography (PET) scans showed bilateral hilar lymphadenopathy and ruled out malignancy. Serum angiotensin-converting enzyme was three times the normal range while calcitriol levels were inappropriately raised, suggesting sarcoidosis as the likely aetiology of the hypercalcaemia. The patient was very responsive to steroid therapy, with serum calcium dropping to normal levels over a four-week admission. The patient developed hypocalcaemia within weeks of discharge, eventually requiring the recommencement of his vitamin D replacement, which has been titrated to maintain normal serum calcium levels.

This case highlights a rare occurrence of two infiltrative disorders with simultaneous differential adverse effects on calcium metabolism, leading to a series of acute hospitalisations often alternating between severe hypocalcaemia or severe hypercalcaemia.

## Introduction

Calcium homeostasis in the human body is regulated by hormones, including parathyroid hormone (PTH) and vitamin D3, which primarily target bones, the gastrointestinal tract, and the kidneys [1]. Calcium levels in the intra and extracellular fluid compartments are tightly controlled by homeostatic mechanisms with serum calcium levels maintained between approximately 2.2-2.5 mmole/L [2]. In this case report, we describe a 73-year-old man with chronic hypoparathyroidism who developed persistent hypercalcaemia due to a likely diagnosis of sarcoidosis.

In health, when a decrease in extracellular calcium is sensed by the parathyroid gland, PTH is released, which in turn acts on the kidneys to increase the activation of vitamin D as well as calcium reabsorption from the nephron [3]. In a hypoparathyroid state, as was the case with our patient, hypocalcaemia occurs as these homeostatic processes are interrupted, resulting in reduced circulating activated vitamin D levels. Vitamin D3 is a steroid hormone critical for intestinal absorption of calcium. It freely undergoes 25 hydroxylation by the liver but only becomes active when further hydroxylated in the kidney to form 1,25(OH)2D. Thus, active forms of vitamin D3 are often used to treat hypoparathyroidism-induced hypocalcaemia.

Hypercalcaemia in patients with treated chronic hypoparathyroidism can occur as a consequence of vitamin D intoxication [4]. In the absence of iatrogenic aetiology, other causes of hypercalcaemia, such as malignancy or granulomatous disease, must be investigated [5]. This case report highlights the complexities of calcium homeostasis and how the dysfunction of its regulators affects serum calcium levels.

## Case presentation

Presentation

A 73-year-old male was admitted to our endocrinology service after transfer from a neighbouring hospital. He presented with lethargy, confusion, generalized weakness, polyuria, and polydipsia. His admission bloodwork showed a serum calcium level of 3.22 mmol/L. Thus, he was diagnosed with symptomatic hypercalcemia prior to his transfer to our care.

Medical history

His medical history includes a distant occurrence of hypoparathyroidism (deemed due to alcohol excess), first diagnosed 10 years prior, for which he was treated with daily alfacalcidol. He also had a history of haemochromatosis that was first diagnosed in 2013, during which his ferritin was elevated at 3821 ng/ml (normal range 24-336 ng/ml). He was found to be homozygous for the *HFE* gene on genetic testing. He later developed deranged liver function tests and bilateral knee pain, which led to biopsies of his liver and synovium that were positive for iron deposition. He was treated with venesections from 2013 to 2017, with ferritin levels decreasing from 3821 ng/ml in 2012 to 46 ng/ml in 2017. His most recent ferritin level three months prior to this admission was 531 ng/ml. Parkinson's disease, osteoarthritis, pseudogout, and a bilateral knee replacement make up the rest of his medical history. He was an ex-smoker.

A year ago, in 2023, the patient had a prolonged stay in another hospital due to symptomatic hypercalcemia. He presented with a six-week history of worsening confusion with a serum calcium of 3.29 mmol/L and magnesium of 0.60 mmol/L on admission. His vitamin D supplementation was stopped during this admission while he was treated with intravenous fluids. His serum calcium returned to within normal limits after five days. He was investigated for malignancy with a CT thorax, abdomen and pelvis, which showed no evidence of a neoplasm, but mediastinal and hilar lymphadenopathy were noted. On discharge, alfacalcidol was recommenced at a lower dose of 0.25 micrograms daily from 1 microgram twice daily prior to admission.

Five months later, he re-visited the same hospital with similar symptoms of hypercalcaemia with an admission serum calcium of 3.83 mmol/L and magnesium of 0.67 mmol/L. He underwent a CT brain for acute confusion that did not show any acute abnormality. A CT scan of thorax, abdomen and pelvis was repeated to outrule malignancy in view of hypercalcemia. The CT report was similar to his scan from the previous year: stable mediastinal and hilar lymphadenopathy, and stable pulmonary nodules without any evidence of malignancy. He received intravenous (IV) zolendronate and was treated with IV fluids. He was discharged after a six-day hospital stay with a serum calcium of 2.48 mmol/L and a presumptive diagnosis of vitamin D over-replacement. The patient’s alfacalcidol and cholecalciferol were stopped permanently on discharge.

Investigation and treatment

Blood test results are shown in Table 1.

**Table 1:** Blood test results performed at the neighbouring hospital (two months before presenting to our hospital) PTH (Parathyroid hormone), 25 OH vitamin D3 (25 Hydroxyvitamin D3), TSH (Thyroid-stimulating hormone), T4 (Thyroxine), ESR (Erythrocyte sedimentation rate).

Parameters	Results	Normal range
Serum calcium	3.83 mmol/L	2.10-2.62 mmol/ L
Serum phosphate	1.30 mmol/L	0.8- 1.5 mmol/ L
Alkaline phosphatase	85 U/L	48-135 U/L
PTH	<1 ng/L	12-88 ng/L
25 OH vitamin D3	57 nmol/L	>50 nmol/L- adequate
Serum creatinine	153 umol/ L	Baseline 100-110 umol/ L
Albumin	37 g/ L	35-52 g/ L
Magnesium	0.67 mmol/ L	0.7- 1.0 mmol/L
TSH	2.24 mlU/ L	0.38- 5.33 mlU/ L
Free T4	13.2 pmol/ L	8-16 p mol/L

Within two months, the patient further deteriorated clinically, leading to his admission to our hospital with symptomatic hypercalcemia of 3.22 mmol/l. The remainder of his bloodwork is shown in Table 2.

**Table 2:** Blood test results at the time of admission PTH (Parathyroid hormone), 25 OH Vitamin D3 (25 Hydroxyvitamin D3), TSH (Thyroid-stimulating hormone), T4 (Thyroxine), ESR (Erythrocyte sedimentation rate), IG (Immunoglobulin), ACE (Angiotensin-converting enzyme).

Parameters	Results	Reference range
Serum calcium	3.22 mmol/l	2.10-2.65 mmol/L
Serum phosphate	1.03	0.8-1.5 mmol/L
Alkaline phosphatase	62 u/L	40-130 u/L
Magnesium	0.66 mmol/L	0.70-1 mmol/L
Albumin	40g/L	335-52 g/L
Creatinine	149 umol/L	100-110 umol/L basline
25 OH vitamin D3	35 nmol/L	>50 – adequate
Serum ACE	217 U/L	6-65 U/L
PTH related peptide	<1.4 pmol/L	0.1-1.4pmol/L
1.25 Di-OH vitamin D (calcitriol)	286 pmol/L	48-192 pmol/L
IgG	25 g/L	6-16 g/L
IgA	1.73 g/L	0.8-4 g/L
IgM	3.35g/L	0.5-2 g/L
24-hour urinary calcium	8.1 mmol/24 hr	2.5 - 7.5 mmol/24 hr
Serum electrophoresis	Raised alfa-2 globulin	
Urine light chains	Negative	
Serum kappa/lambda chain ratio	1.13	0.26-1.65

The patient’s two previous admissions were reviewed for the investigations of his hypercalcaemia to date. The consistent findings of hilar and mediastinal lymphadenopathy were of note. Serum angiotensin-converting enzyme (ACE) was measured at three times the upper limit of the normal range. Additionally, calcitriol (1,25 dihydroxy vitamin D) levels were inappropriately high.

**Figure 1:**
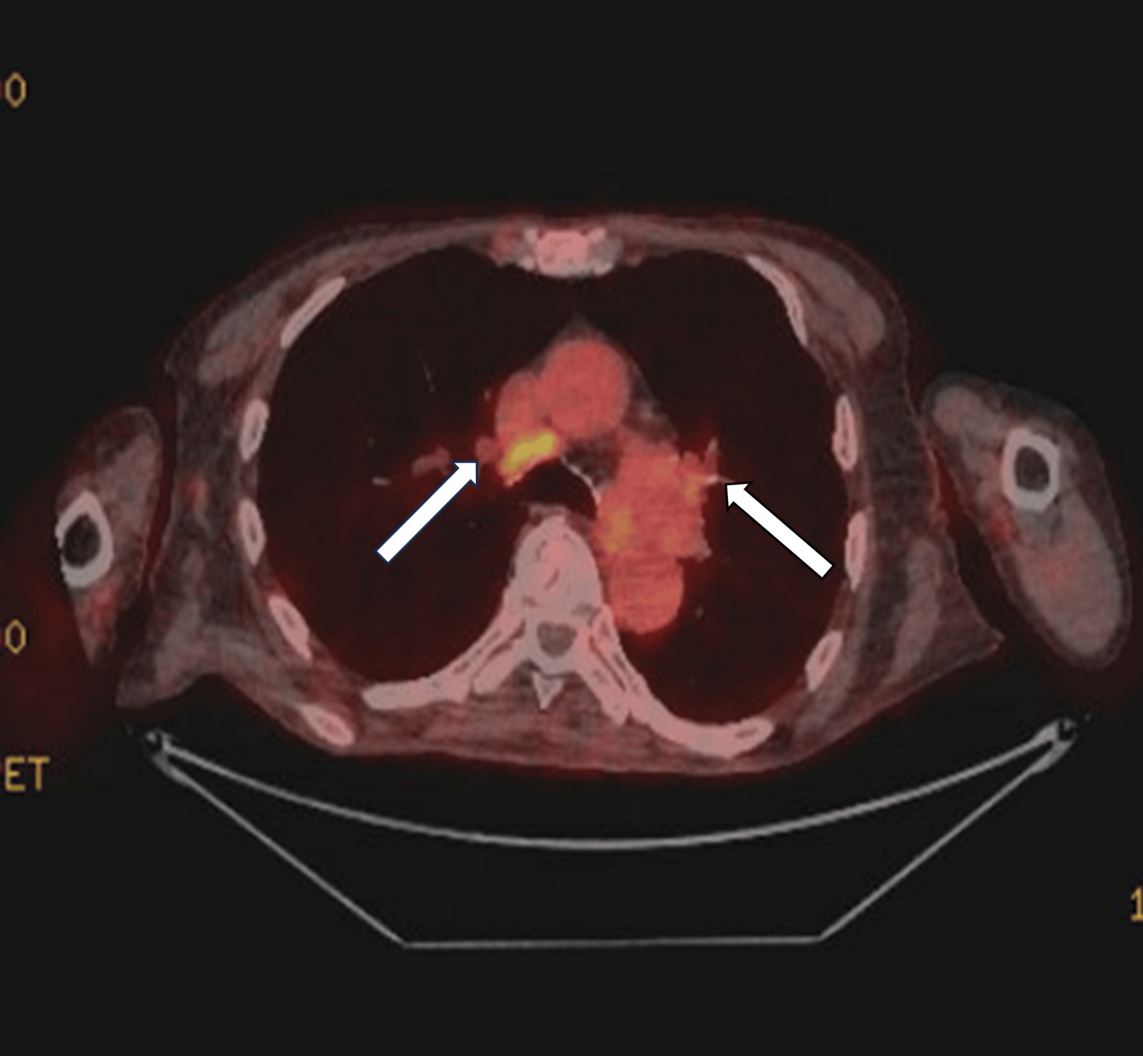
PET CT scan image Mildly fluorodeoxyglucose (FDG) avid bilateral hilar lymphadenopathy was seen. PET: Positron emission tomography.

To rule out malignancy or paraneoplastic syndrome aetiology, a positron emission tomography (PET) scan was performed and parathyroid hormone (PTH)-related peptide was measured. The full-body PET scan showed no signs of malignancy but was reported as “stable mediastinal and hilar lymphadenopathy likely benign aetiology with sarcoidosis as potential aetiology” (Figure 1). Due to the increasing suspicion of sarcoidosis, a biopsy through bronchoscopy was discussed with the patient, but he did not give his consent for the procedure.

The patient was treated with intravenous fluids on admission, and then glucocorticoids were commenced with for likely sarcoidosis. Over the course of a four-week hospital stay, the calcium levels decreased to 2.65 mmol/l before discharge with continuing steroid treatment.

Follow up

Two weeks later, he returned for a follow-up appointment. He was hypocalcaemic at 1.90 mmol/l but was asymptomatic. His phosphate was 0.88 mmol/L and magnesium 0.56 mmol/l. Magnesium and calcium were repleted and steroids held. He was discharged on a reduced dose of steroids with further follow-up blood tests due in one week.

The following week, he presented as asymptomatic with a serum calcium of 1.43 mmol/l, phosphate level of 1.11 mmol/L, and magnesium level of 0.61 mmol/l. He was admitted and treated similarly to his most recent admission. On discharge, his alfacalcidiol was restarted at a dose of 0.25 mg daily. He would present two weeks afterward hypocalcaemic at 1.80 mmol/L. His dose of alfacacidiol was increased to 0.50 mg once daily at this time. A trend of laboratory values, including serum calcium, throughout his hospital admissions and at follow-up is demonstrated in Table 3. He is followed up for titration of his alfacalcidol based on his serum calcium levels. He is currently on alfacalcidol 0.25 mcg daily and has maintained serum calcium levels within a normal range for several months.

**Table 3:** Trends throughout admissions Admission 1 - Neighbouring hospital, two months before presenting to our hospital; Admission 2 - First admission to our hospital; Admission 3 + 4 - Re-admissions for treatment after hypocalcaemia noted at follow-up.

	Admission No.1	Admission No.2	Admission No.3	Admission No.4
Serum calcium (mmo/L)	3.83	3.22	1.90	1.43
Serum phosphate (mmol/L)	1.30	1.03	0.88	1.11
Alkaline phosphatase (u/L)	85	62	60	73
PTH (ng/L)	<1			<4
25 OH vitamin D3 (nmol/L)	57	35	27	33
Serum creatinine (umol/L)	153	149	154	112
Albumin (g/l)	37	40	42	38
Protein electrophoresis	Raised alfa-2 globulin consistent with mild inflammation			

## Discussion

This case report describes a patient with chronic hypoparathyroidism who subsequently developed hypercalcaemia that persisted despite discontinuation of vitamin D supplementation. Investigation of this hypocalcaemia revealed granulomatous disease, namely sarcoidosis, to be the probable cause. The aetiology of the patient’s longstanding hypoparathyroidism was deemed due to alcohol excess at diagnosis in 2012. The chronicity of his disease is not typical of alcohol-induced hypoparathyroidism, which tends to be transient, occurring due to alcohol-driven magnesium deficiency, which subsequently causes a reduction in PTH excretion. Though the patient was slightly hypomagnesaemic during multiple admissions, it was never severe (0.4 mmol/L or lower) [6]. His ferritin levels at the time of his hypoparathyroidism diagnosis, in addition to the iron deposition found in his liver and synovium, were highly suggestive of iron overload as the probable aetiology of his hypoparathyroidism.

Hypercalcaemia in patients with chronic hypoparathyroidism has been described in several case reports [7,8]. In the majority of cases, the aetiology of this hypercalcaemia is due to over-supplementation of Vitamin D. Over-treatment of the patient’s hypoparathyroidism was thought on multiple admissions to be the cause of his hypercalcaemia. His alfacalcidol dose was decreased and then ultimately stopped as his hypercalcaemia persisted. A de novo cause for the hypercalcaemia had to be considered as the hypercalcaemia persisted despite stopping the vitamin D supplementation. CT scans and the whole-body PET scan showed no evidence of malignancy, while PTH-related peptide levels were not elevated.

Though a tissue diagnosis could not be made, the combination of hypercalcaemia, increased serum ACE levels and bilateral hilar lymphadenopathy (seen on CT and PET scans) are highly suspicious for sarcoidosis. Hypercalcaemia develops in approximately 10% of sarcoidosis cases [9]. It is caused by macrophage-mediated 1 alpha hydroxylation of vitamin D3, causing increased production of 1,25(OH)2D3 (calcitriol), which is usually tightly controlled in healthy subjects. Calcitriol levels tend to be inappropriately normal or even increased in sarcoidosis, and it was present in this case [10].

Hypercalcaemia secondary to sarcoidosis is primarily treated with corticosteroid therapy. The British Thoracic Society recommends initial treatment with prednisolone 10-40mg daily with tapering if a good clinical response is noted [11]. Glucocorticoids treat hypercalcaemia in granulomatous disease by reducing the amount of circulating calcitriol through macrophage 1 hydroxylation inhibition [12].

The challenge of managing the patient’s serum calcium levels centered on his dual diagnoses of hypoparathyrodism and probable sarcoidosis, each causing calcium homeostasis dysfunction in different ways and in close temporal proximity, resulting in a complex clinical challenge. Initial presentations with hypercalcaemia were attributed to his alfacalcidol treatment, leading to its discontinuation. The diagnosis of granulomatous-driven hypercalcaemia was finally arrived upon due to the persistence of symptomatic hypercalcaemia. The excellent response to steroid therapy unmasked the patient’s chronic hypoparathyroidism, which is likely irreversible due to long-standing haemochromatosis. 

In retrospect, perhaps a block-and-replace method would have been useful whereby steroids and alfacalcidol would be given simultaneously, blunting the hypercalcaemia of the granulomatous disease while preventing the hypocalcaemia that returned in this case. For example, 30 mg prednisolone with 0.025 mg alfacalcidol daily with weekly follow-up and tapering of steroids once calcium is in the normal range.

## Conclusions

In summary, we present a case of a 73-year-old man with chronic hypoparathyroidism due to previously uncontrolled haemochromatosis who developed hypercalcaemia. In cases of treated hypoparathyroidism, vitamin D intoxication is the most common cause of this presentation. After ruling this out, further investigations lead to a likely diagnosis of sarcoidosis. Thus, this case highlights a rare occurrence of two infiltrative disorders with simultaneous differential adverse effects on calcium metabolism, leading to a series of acute hospitalisations often alternating between severe hypocalcaemia or severe hypercalcaemia.
